# Supramarginal Resection of Metastatic Brain Tumors: A Meta-Analysis Study

**DOI:** 10.3390/medicina61081446

**Published:** 2025-08-12

**Authors:** Florin Adrian Tofan, Ahmed T. Massoud, Cosmin Ioan Faur, Stefan Ioan Florian

**Affiliations:** 1 Department of Neurosurgery, Faculty of Medicine, University of Medicine and Pharmacy Iuliu Hatieganu, 400012 Cluj Napoca, Romania; 2 Faculty of Medicine, Fayoum University, Fayoum 63514, Egypt; 3 Regina Maria Dental Departament, Regina Marina Private Healthcare Network, 014416 Bucharest, Romania

**Keywords:** gross total resection, supramarginal resection, brain metastases

## Abstract

*Background and Objectives*: Over 30% of people who suffer from cancers are at risk of developing brain metastases. The typical recommended surgical therapy for metastases within the brain is gross total resection (GTR). Nevertheless, GTR solely may not always be adequate for disease management since remaining tumors can show local advancements and invasion. The focus of this research is to summarize the current data and to compare the outcomes of GTR and supramarginal resection. *Materials and Methods*: A search on the PubMed, Scopus, Cochrane Central Library, and Web of Science (WOS) databases was performed using specific keywords for single or multiple brain metastasis of any origin in patients who underwent either supramarginal resection or gross total resection. *Results*: The average age of the patients involved in the study spanned between 51 ± 6 years and 60.5 ± 10.1 years. Males represented 48.7% of the total population. The incidence of 1-year survival among the GTR group was 37.1%, whereas the supramarginal resection group showed an incidence of 91.3%, under the random effect model (0.551, 95% CI [0.18, 0.921]). The incidence of 2-year survival among the GTR group was 21.26%, whereas the supramarginal resection group showed an incidence of 72.46%, under the random effect model (0.380, 95% CI [0.113, 0.648]). The incidence of local recurrence among the GTR group was 57.69%, whereas the supramarginal resection group showed an incidence of 18.4%, under the random effect model (0.266, 95% CI [0.106, 0.426]). *Conclusions*: Supramarginal resection is a promising approach for the management of brain metastases.

## 1. Introduction

It was estimated that over 30% of people who suffer from cancers are at risk of developing brain metastases [1]. The past four decades have shown a rising incidence of brain metastases that fluctuated between 5% and 40% [2,3]. Several therapeutic choices are accessible for cancer patients, including surgical resection. Yet, the optimal surgical approach necessitates a personalized approach as patient-specific factors such as age, co-morbidities, the mass size, primary tumor type, and metastatic burden could influence their outcomes [4,5]. The other therapeutic options include radiotherapy and chemotherapeutic agents. However, monotherapy with one of these approaches is not enough for controlling disease progression. Thus, management of brain metastases usually encompasses multiple therapeutic options [6,7,8,9,10].

Several factors were found to affect the outcomes of therapy, with no consensus regarding the extent of the surgery. The brain tissue has been thought to provide a clear definition of brain metastases with no extension beyond the tumor margins. Consequently, the typical recommended surgical therapy for metastases within the brain is gross total resection (GTR). Nevertheless, GTR solely may not always be adequate for disease management since remaining tumors can show local advancements and invasion [11,12]. According to reports, the incidence of local tumor growth following GTR might reach up to 53% without radiotherapy [13]. Furthermore, 10% to 34% of patients had a relapse within the postoperative area 1 year after the operation, regardless of whether they received radiotherapy or not [14,15].

The concept of supramarginal resection was first proposed in 2009 by Yoo et al. The supramarginal resection approach was hypothesized for the removal of invisible malignant cells within the surrounding 5 mm of brain parenchyma. Thus, excision of the whole metastatic lesion was supposed to minimize recurrence [16]. However, the applicability of this approach must be balanced against individual patient factors, including lesion location (eloquent vs. non-eloquent area) and goals of care, emphasizing personalized surgical planning [5].

There are variable incidences reported in the literature about the survival and local recurrence in patients who underwent surgical resection via the GTR approach or supramarginal approach. Consequently, the focus of this research is to summarize the current data and compare the outcomes of GTR and supramarginal resection.

## 2. Materials and Methods

Study design:

The documentation of this systematic review and meta-analysis was based on the 2020 statement of the Preferred Reporting Items for Systematic reviews and Meta-Analyses (PRISMA) checklist guidelines [17]. In addition, we adhered to the methodology recommendation of the “Cochrane Handbook for Systematic Reviews and Meta-Analyses” (London, UK) [18].

Eligibility criteria:

Our population of interest was patients with single or multiple brain metastases of any origin, who underwent either supramarginal resection or gross total resection since 2014. Observational studies or trials that reported survival outcomes were included.

We excluded studies that included patients with only subtotal resection, or studies that reported outcomes of surgical resection without subgrouping according to the extent of the resection. In addition, conference abstracts, letters to the editor, case reports, and editorials were excluded.

Literature search:

We searched the electronic databases of PubMed, Scopus, Cochrane Central Library, and Web of Science (WOS). The following search strategy was applied for all the databases (“Brain Metastasis” OR “brain metastases” OR “metastatic brain tumors”) AND (“supratotal resection” OR “supramaximal resection” OR “supracomplete resection” OR “FLAIR resection” OR “lobectomy” OR “supramarginal resection” OR “gross total resection”). The search results were restricted to studies published since 2014.

Study selection and Data extraction:

The search results were imported into EndNote software version X9 to remove the duplicated articles. The remaining articles were exported and uploaded into a Microsoft Excel 2019 spreadsheet. Two authors separately reviewed all the articles for eligibility according to the previously stated eligibility criteria.

A two-step screening process was employed: first, the titles and abstracts were reviewed, and then the full-text articles of potentially eligible studies were assessed for final inclusion. A senior author provided a final independent review to resolve any disagreements between the reviewers.

We extracted the data from each study, including the first author’s last name, country, year of publication, study design, sample size, procedure, post-operative complications, and demographic details (such as sex and age). In addition, we extracted the results of 1year of overall survival, 2 years of overall survival, and the incidence of local recurrence of brain metastases.

Quality assessment and Risk of bias:

The New Castle Ottawa scale (Newcastle, Australia and Ottawa, ON, Canada) was used to assess the quality of the included cohorts. It has three key domains: selection, comparability, and outcome. Thresholds for translating the NOS score were determined according to the Agency for Healthcare Research and Quality (AHRQ) (North Bethesda, MD, USA). For judgment of good quality: 3 or 4 stars in the selection domain AND 1 or 2 stars in the comparability domain AND 2 or 3 stars in the outcome/exposure domain. For judgment of fair quality: 2 stars in the selection domain AND 1 or 2 stars in the comparability domain AND 2 or 3 stars in the outcome/exposure domain. For judgement of poor quality: 0 or 1 star in the selection domain OR 0 stars in the comparability domain OR 0 or 1 stars in the outcome/exposure domain [19]. Discrepancies in assessment were resolved by consensus following a discussion with a senior author. According to Egger et al., publication bias could not be assessed since no outcome included at least 10 studies [20].

Statistical analysis:

The statistical analyses were conducted using Open Meta-analyst software, version 4. Subgroup analyses were performed according to the procedure undertaken, whether supramarginal resection or GTR.

The extracted data were pooled as odds ratio and 95% confidence intervals (CI). A forest plot approach was used for better visualization of data. A random-effect meta-analysis model was applied since there was apparent clinical heterogeneity in the included studies due to variations in intervention protocols, origin of metastases, and number of metastases.

## 3. Results

**Figure 1:**
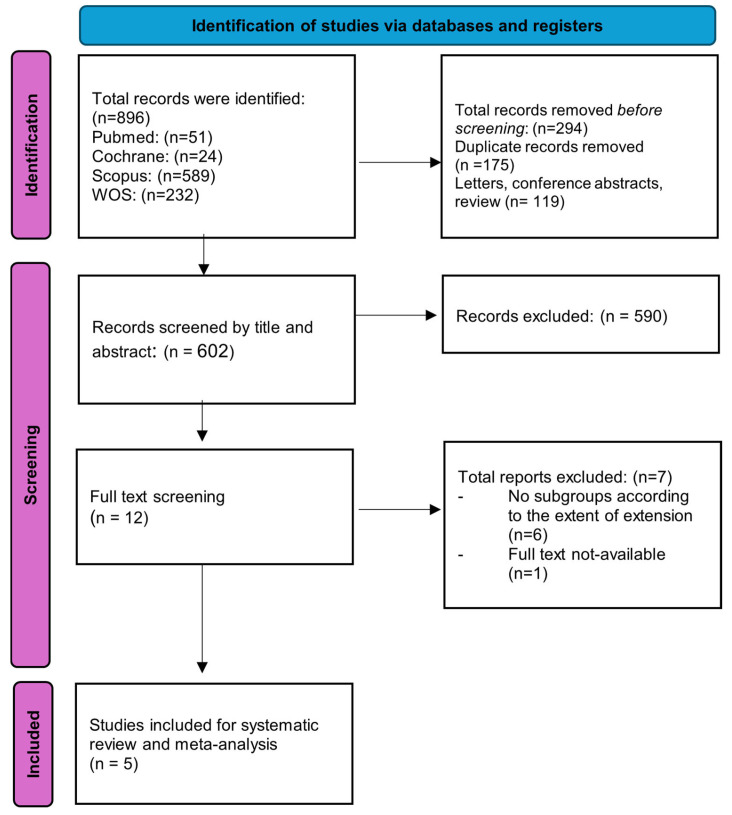
Selection process of the eligible studies.

Literature search:

An initial database search from 2014 till now retrieved 896 results (Figure 1). After the removal of duplicated references, reviews, letters, and conference abstracts, 602 articles remained for screening by title and abstract. A total of 12 articles were passed for full-text screening. Eventually, five studies were included for systematic review and meta-analysis.

Studies summary and baseline characteristics:

The eligible studies were retrospective cohorts having a total sample size of 499 patients (Table 1). The studies showed variable geographical distribution due to the different continents on which the studies were performed. Two studies included patients with single brain metastases, whereas the remaining three studies included patients with single or multiple brain metastases [21,22]. The origin of the metastases was predominately from lung cancers [23,24,25].

The average age of the patients involved in the study spanned between 51 ± 6 years and 60.5 ± 10.1 years (Table 2). Males represented 48.7% of the total population. Two studies included patients who underwent GTR, two included patients who underwent supramarginal resection, and only one study compared both procedures.

Quality assessment:

The selection domain showed no possible bias among the included studies. Comparability was based on age and sex, which were accounted for in all of the included studies. In addition, the follow-up and outcome assessments were judged as having no potential risk of bias. Thus, all the studies verified the needed criteria for being judged by the authors as good quality (Table 3).

Outcomes

**Figure 2:**
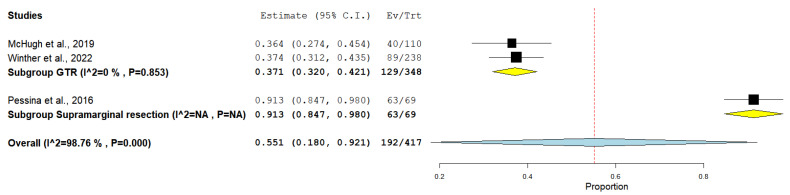
Forest plot of 1-year overall survival outcome [21,22,25].

1-year overall survival:

The rate of 1-year overall survival was documented in two studies that performed GTR and one study that performed supramarginal resection. Collectively, the GTR group included 348 patients, and the supramarginal resection group included 69 patients (considering surgical reintervention, Figure 2).

The incidence of 1-year survival among the GTR group was 37.1%, whereas the supramarginal resection group showed an incidence of 91.3%, under the random effect model (0.551, 95% CI [0.18, 0.921]). The overall studies showed heterogeneity, which was resolved using subgroup analysis (*p* = 0.853).

**Figure 3:**
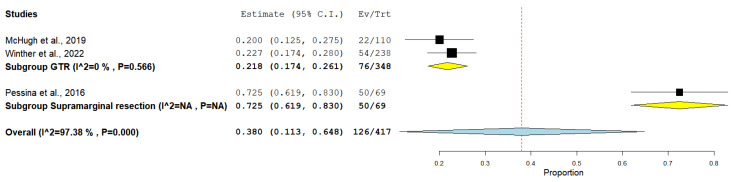
Forest plot of 2-year overall survival outcome [21,22,25].

2-year overall survival:

The rate of 2-year overall survival was documented in two studies that performed GTR and one study that performed supramarginal resection. Collectively, the GTR group included 348 patients, and the supramarginal resection group included 69 patients (considering surgical reintervention, Figure 3).

The incidence of 2-year survival among the GTR group was 21.26%, whereas the supramarginal resection group showed an incidence of 72.46%, under the random effect model (0.380, 95% CI [0.113, 0.648]). The overall studies showed heterogeneity, which was resolved using subgroup analysis (*p* = 0.566).

**Figure 4:**
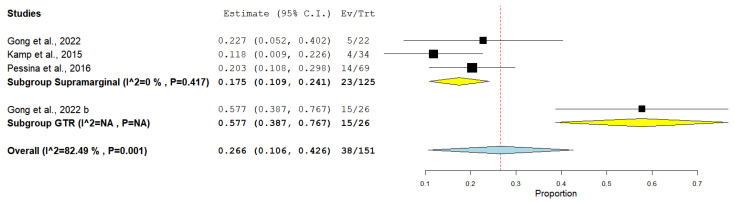
Forest plot of the incidence of local recurrence of brain metastases [21,23,24].

Local recurrence of brain metastases:

The rate of local recurrence after 1 year from the operation of brain metastases was documented in three studies that performed supramarginal resection and one study that performed GTR. Collectively, the GTR group included 26 patients, and the supramarginal resection group included 125 patients (Figure 4).

The incidence of local recurrence among the GTR group was 57.69%, whereas the supramarginal resection group showed an incidence of 18.4%, under the random effect model (0.266, 95% CI [0.106, 0.426]). The overall studies showed heterogeneity, which was resolved with subgroup analysis (*p* = 0.417).

## 4. Discussion

To our knowledge, this is the first meta-analysis summarizing the results of supramarginal resection and GTR among patients with brain metastases. The extent of resection of brain metastases is a critical point that should be addressed carefully, since incomplete surgical resections may contribute to a high risk of local brain recurrence. On the other hand, extensive resection carries the risk of permanent neurological injury [5].

The overall survival was assessed in this study at 1 and 2 years. The incidence of 1-year survival among the GTR group was 37.1%, whereas the supramarginal resection group showed an incidence of 91.3%. The incidence of 2-year survival among the GTR group was 21.26%, whereas the supramarginal resection group showed an incidence of 72.46%. Despite the decreased incidence of survival with the progression of time, supramarginal resection showed more favorable survival outcomes. Pessina et al. reported an average overall survival time of 24 months for patients who were treated by supramarginal resection [21]. In contrast, Yoo et al. reported a higher average survival duration for the GTR group. They found that the GTR group showed an average survival of 10.3 months and the supramarginal resection group showed an average survival duration of 11 months (*p* = 0.54). However, in agreement with our results, the incidence of survival was significantly higher among the supramarginal resection group (27.3%), in comparison to the GTR group (3.8%, *p* = 0.001) [16].

In this research, we discovered that the incidence of local recurrence after one year from the operation among the GTR group was 57.69%, whereas the supramarginal resection group showed an incidence of 18.4%. In agreement with our results, Yoo et al. have reported that the incidence of recurrence was significantly lower among patients who were treated by supramarginal resection (23.3%), as compared to the incidence of survival among the GTR group (43.1%, *p* = 0.04) [16]. This was also consistent with the results reported by Gong et al. where they noted that the recurrence rate after 1 year from the operation was significantly elevated among patients who underwent GTR (57.7%) relative to patients who underwent supramarginal resection (22.7%, *p* = 0.014). Furthermore, they observed that the average survival time without further advancement in the brain metastases was 7 months among the GTR group compared to 14 months among the supramarginal resection group [23]. Patients in the study by Gong et al. did not receive any adjuvant therapeutic agent for cancers, which indicates that the survival outcomes are solely attributed to the surgical procedure. The lower recurrence rate among the supramarginal resection group might be explained by the wider resected area, which leaves a lower probability for remaining cancerous cells within the site of the metastases. It was evident in a previous study that 34.7% of malignant cells invade the local brain tissue surrounding the metastatic lesion [26]. Two earlier research articles examined several autopsies of patients with brain metastases and detected residual tumors among 25% to 42% of patients who were surgically treated [27,28]. Thus, widening the extent of resection to about 5mm beyond the margin might be associated with better outcomes of recurrence and survival [16,23].

However, there are concerns regarding the neurological deficit that could appear, especially when the lesion is in an eloquent area. Kamp et al. observed neurological deficits in 14.7% of the patients undergoing supramarginal resection in eloquent areas [24]. Yet Yoo et al. found that supramarginal resection controlled the local recurrence even without radiotherapy; however, consistent with Gong et al. and Pessina et al., they excluded patients having tumors in eloquent areas from the supramarginal resection arm [16,21,23]. Instead, those patients underwent GTR. On the other hand, Kamp et al. were the first to investigate the outcomes of supramarginal resection in eloquent brain tumors. They removed only 3 mm of the brain tissue around the tumor, along with intraoperative neuromonitoring. Although some patients had neurological complications, these resolved in the following days [24]. Indeed, it has been shown that performing tumor resection while the patients are awake is associated with lower rates of neurological complications even in eloquent areas [29]. This is important since resection of tumors in eloquent areas is associated with increased risk of neurological deficits and worse survival [30,31]. These findings highlight that supramarginal resection benefits should be weighed against location-specific risks, necessitating tailored surgical planning.

Beyond the tumor location in the brain, several other factors could affect the outcomes of patients undergoing resection of metastatic brain tumors, including the stage of cancer, patients’ age and co-morbidities, the number of lesions, and the primary tumor type [4,32]. For instance, it has been shown that the metastatic burden was associated with the survival rate rather than the resection area [33]. Also, some concerns were raised regarding the number of foci and their location. For instance, Sebastian et al. showed that multiple brain foci resection resulted in long-term neurological complications in 10.6% of the patients, which is associated with worse survival outcomes. Furthermore, the tumor site in critical areas was associated with higher rates of complications [32]. Thus, the decision of the surgical approach should be carefully tailored according to the patient’s condition.

Particularities and future perspectives

The main strength of this meta-analysis is that it is the first to summarize the current literature regarding the management of brain metastases with supramarginal resection or GTR. Subgroup analysis provides homogeneous data, which makes the results of this meta-analysis more reliable. However, one important limitation is the small number of studies in the literature that study the effect of the extent of resection on survival and local recurrence outcomes. Consequently, the number of the included studies is low, which restricts the statistical power of our analysis and the generalizability of the findings. In addition, the observational nature of the included studies adds a limitation to this study. A critical limitation is the risk of selection bias from indirectly comparing non-randomized cohorts. Surgeons likely performed GTR on patients with higher-risk tumors (e.g., in eloquent areas), while reserving supramarginal resection for more favorable cases. This confounding by indication means the superior outcomes we observed for supramarginal resection may reflect this patient selection rather than the technique’s efficacy, highlighting the need for randomized controlled trials.

Our study provided evidence on the efficacy of supramarginal resection in patients with metastatic brain tumors. Patients undergoing supramarginal resection had higher survival rates and lower rates of recurrence compared to those undergoing gross total resection. However, this should be interpreted with caution since the evidence is from retrospective observational studies. Thus, randomized clinical trials are needed to provide high-quality evidence comparing supramarginal resection and GTR. Furthermore, studies should stratify outcomes by primary tumor origin (e.g., lung, breast, melanoma), metastasis burden, and lesion site. Additionally, the effect of supramarginal resection along with different treatment modalities needs further investigation. Considering that multiple factors could affect the outcomes of supramarginal resection, the choice of undergoing this approach should be carefully individualized according to each case.

## 5. Conclusions

Supramarginal resection is a promising approach for the management of brain metastases. It was associated with an improved local recurrence incidence as well as better 1- and 2-year overall survival rates when compared to GTR. However, careful patient selection should take place based on the potential risks and benefits of each case when selecting this technique.

## Figures and Tables

**Table 1:** Summary of the characteristics of the included studies.

Author and Year	Country	Study Design	Sample Size	Primary Tumor	Number of Metastases
Gong et al., 2022 [23]	China	Retrospective cohort	48	Lung adenocarcinoma	Single or multiple
Kamp et al., 2015 [24]	Germany	Retrospective cohort	34	Adenocarcinoma 25 (73.5%) Small cell cancer 2 (5.9%) Clear cell cancer 1 (2.9%) Squamous cell cancer 1 (2.9%) Malignant melanoma 5 (14.7%)	Single or multiple
McHugh et al., 2019 [25]	New Zealand	Retrospective cohort	110	Melanoma (100%)	Single or multiple
Pessina et al., 2016 [21]	Italy	Retrospective cohort	69	Breast cancer 24 (34.8%) Non-small cell lung cancer 21 (30.4%) Melanoma 15 (21.7%) Other (clear cell carcinoma, colon) 9 (13.1%)	Single
Winther et al., 2022 [22]	United States	Retrospective cohort	238	Lung 79 (33%) Melanoma 59 (25%) Colorectal 27 (11%) Breast 22 (9%) Kidney 13 (5%) Other 26 (12%) Unknown origin 12 (5%)	Single

**Table 2:** Summary of study groups, patients’ demographics, and postoperative complications.

Author and Year	Study Groups	Age	Sex (Males%)	Postoperative Complications
Gong et al., 2022 [23]	Supramarginal	60.5 ± 10.1	11 (50%)	NR
	GTR	55.2 ± 11	11 (42.3%)	
Kamp et al., 2015 [24]	Supramarginal	60 ± 12.5	10 (29.4%)	NR
McHugh et al., 2019 [25]	GTR	59.9 ± 10.95	69 (63%)	NR
Pessina et al., 2016 [21]	Supramarginal	51 ± 6	27 (39%)	NR
Winther et al., 2022 [22]	GTR	64 ± 11.8	115 (48%)	Intracranial hemorrhage 7 (3%) Pneumonia or pulmonary embolism 1 (0.4%) Bone flap infection 1 (0.4%) CSF leakage 2 (2%) Intracerebral abscess 2 (2%) Other complications in need of neurosurgical intervention 3 (1%)

Abbreviations: GTR, gross total resection; NR, not reported.

**Table 3:** Quality assessment of the included studies.

Study	Cohort Studies									
	Selection				Comparability	Outcome			Score	Quality
	Representativeness of the Exposed Cohort	Selection of the Non-Exposed Cohort	Ascertainment of Exposure	Demonstration that Outcome of Interest Was Not Present at Start of the Study	Comparability of Cohorts on the Basis of the Design or Analysis: (Age and Sex)	Assessment of Outcome	Was Follow-Up Long Enough for Outcomes to Occur?	Adequacy of Follow-Up of Cohorts		
Gong et al., 2022 [23]	*	*	*	*	**	*	*	*	9	good
Kamp et al., 2015 [24]	*		*	*	**	*	*	*	8	good
McHugh et al., 2019 [25]	*	*	*	*	**	*	*	*	9	good
Pessina et al., 2016 [21]	*		*	*	**	*	*	*	8	good
Winther et al., 2022 [22]	*	*	*	*	**	*	*	*	9	good
